# Supplementary material for: Tensor Hypercontraction Error Correction Using Regression
Source: J Comput Chem. 2026 Mar 17;47(8):e70354. doi: 10.1002/jcc.70354 (PMC12996760; doi:10.1002/jcc.70354)
Supplement: Supplementary file 1 — Data S1. Supporting Information. [file JCC-47-0-s001.pdf]

Supplemental Information for Tensor Hypercontraction Error Correction Using Regression

Ishna Satyarth,[1] Eric C. Larson,[1] and Devin A. Matthews[2]

[1)] *Department of Computer Science, Southern Methodist University, Dallas, TX 75275, USA*

[2)] *Department of Chemistry, Southern Methodist University, Dallas, TX 75275, USA*[a)]

[a)]Electronic mail: {isatyarth,eclarson,damatthews}@mail.smu.edu

S1: KRR Hyper-parameters α and γ

For the KRR-Molecule model, we created separate heatmaps which highlight [α, γ] pairs with maximum RMSE percentage improvement for different δ values as shown in Figure S1. As an example, the heatmap for $\delta = 1.25$ shows that some [α, γ] pairs do not reduce the THC errors; these points in the heatmap have negative improvement values (truncated to zero in the heatmap representation for clarity) and are colored dark blue. Thus, the KRR-Molecule fit at these combinations will not yield a good training result of the regression model. The [α, γ] pairs colored light blue to light green indicate medium performance of error correction. Also, for the [α, γ] pair of values [1e-06, 1e-04], the percentage improvement is the highest value observed at 83%. This indicates that the learning is maximized at this point and the RMSE has been significantly reduced using this combination of hyper-parameters. Also, the value 82% for some combinations, including [1e-06, 1e-05], is very close to 83%, which indicates more than one possible maximal combination of [α, γ] for optimization of the KRR-Molecule model. As the grid search is highly discretized, the true maximum cannot be exactly inferred, nor can the existence of multiple local maxima be distinguished from a single smooth, broad maximum. However, multiple Nelder–Mead optimizations started at different points confirms a single maximum. Hence, we then optimized this smaller range of [α, γ] for calculating the root mean square error through Nelder–Mead optimization using starting point for optimization as [1e-06, 1e-04], and range of α [1e-09, 1e-05] and range of γ [1e-07, 1e-04]. Likewise, we can obtain a smaller range of [α, γ], where we need to optimize the RMSE, for δ=1, 1.5, 1.75 and 2, using the Nelder–Mead optimization.

The trend in the heatmaps in Figure S1 shows that the best fit of KRR-Molecule is possible with lower α and lower γ values, which is upper left corner of the heatmap. Lower α indicates the model is more flexible and captures the complexities of the training data, whereas lower γ means smoother model and a wider influence of each training example. This means that the relationships in the data are relatively simple and the model is less sensitive to noisy data. In summary, lower values for α and γ in KRR-Molecule leads to a more flexible model with potentially stronger regularization against noise.

Likewise, for KRR-ΔMolecule model, we generated the heatmap to analyze the RMSE percentage improvement for δ values 1, 1.25, 1.5, 1.75, 2 in Figure S2. Contrary to the KRR-

TABLE S1. Final KRR Hyper-parameters from Nelder–Mead downhill simplex-optimization.

δ	KRR-Molecule		KRR-ΔMolecule	
	α	γ	α	γ
1	1.40e-08	5.75e-07	1.00e-03	1.28e-02
1.25	1.98e-06	1.02e-04	2.03e-04	7.87e-03
1.5	1.51e-08	5.50e-07	7.50e-10	1.21e-07
1.75	1.21e-09	7.50e-08	1.22e-09	7.25e-08
2	1.15e-08	8.81e-07	1.00e-10	2.63e-08

Molecule model, in the KRR-ΔMolecule model, most of the points or [α, γ] pair have best fit indicated as yellow in the heatmap, which is mostly present all over the heatmap. We identified the value pair of [α, γ] for all the δ correction for both KRR-Molecule and KRR-ΔMolecule models for all δ values, and perform Nelder–Mead downhill simplex optimization next. We see that in KRR-ΔMolecule model, the [α, γ] pair with best fit has lower α and medium range of γ, indicating non-linearity in the dataset. Lower α indicates model is able to capture more complex patterns, and a medium range of γ allows the model to consider a reasonable "neighborhood" of data points for making predictions, balancing model complexity and generalization ability.

The final hyper-parameters obtained from Nelder–Mead downhill simplex optimization for both KRR-Molecule and KRR-ΔMolecule have been recorded in Table S1.

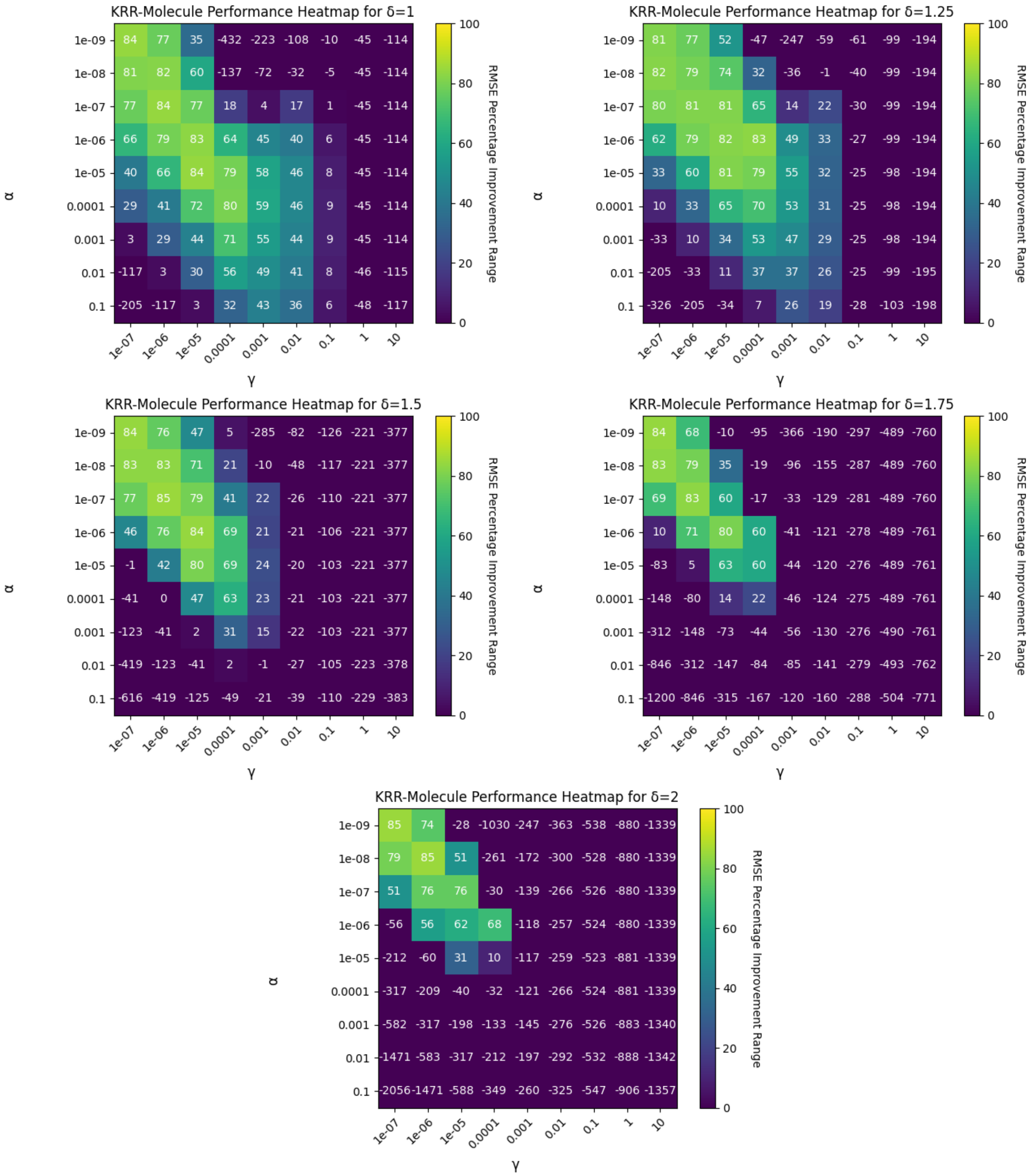

FIG. S1. Heatmap of RMSE %IMP for KRR-Molecule model for δ values 1, 1.25, 1.5, 1.75, 2. Dark blue indicates points that are zero or negative.

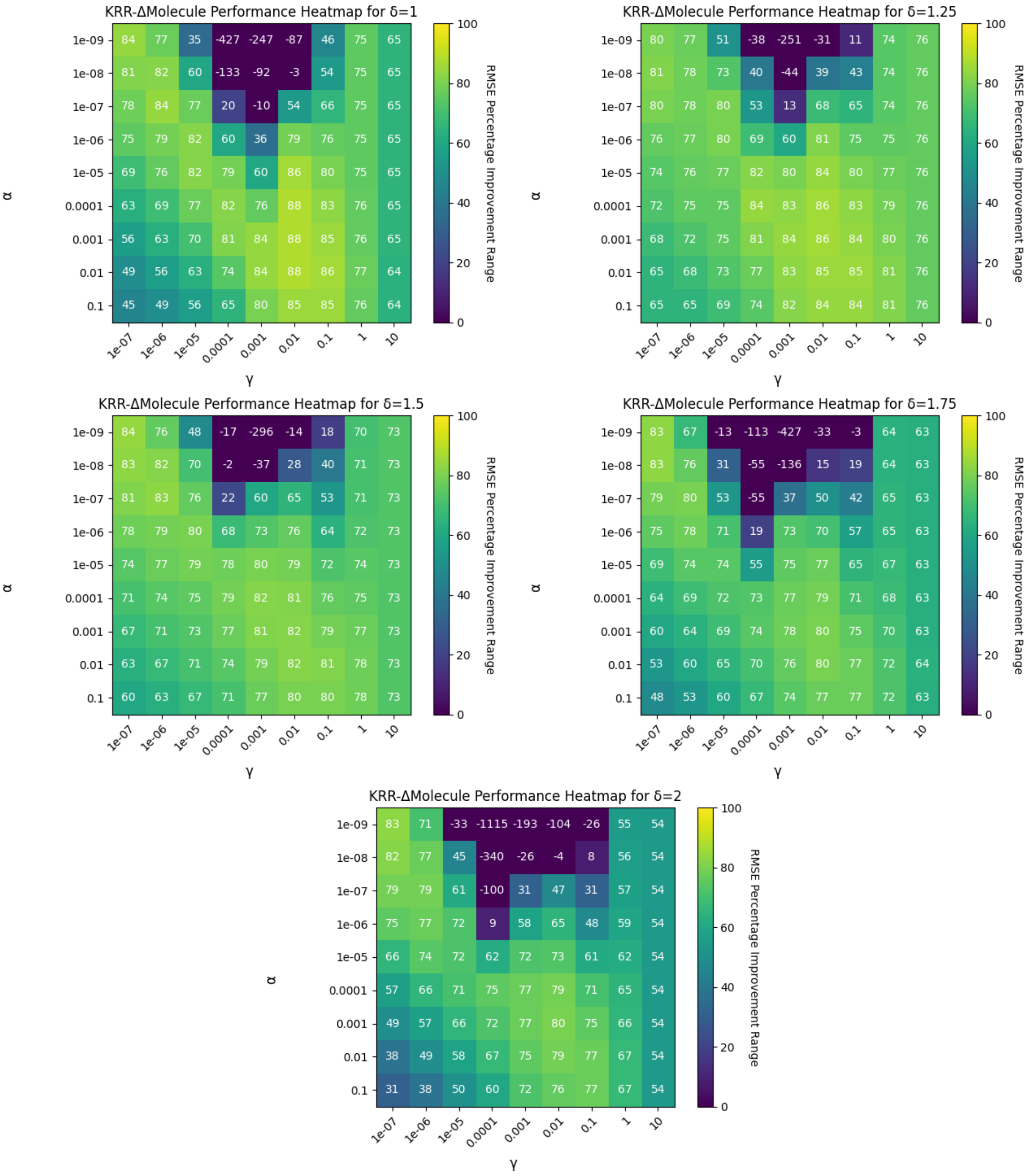

FIG. S2. Heatmap of RMSE %IMP for KRR-ΔMolecule model for δ values 1, 1.25, 1.5, 1.75, 2. Dark blue indicates points that are zero or negative.
